# Managing integration and disintegration: performance conditions of patient and public involvement to hospitals quality improvement

**DOI:** 10.1108/JHOM-10-2024-0410

**Published:** 2026-04-02

**Authors:** Bernard Voz, Benoît Pétré, Jean-François Orianne

**Affiliations:** Public Health Sciences Department, University of Liège, Liège, Belgium; Social Science Research Institute (IRSS), University of Liège, Liège, Belgium

**Keywords:** Hospital, Quality, Patient and public involvement, Patient and family advisory council, Participation performance, Systems theory

## Abstract

**Purpose:**

Patient and public involvement (PPI) has been a major issue for health systems for the last decade. PPI generated great expectations such as more patient-centred health systems and improved quality. However, evidence remains patchy. Most of the literature depicts a lack of evidence on the effectiveness of PPI initiatives. This paper aims to go beyond the performance measurement to analyse the performance conditions of PPI initiatives at the organisational level.

**Design/methodology/approach:**

Building on Luhmann’s systems theory, we approach the performance conditions of PPI initiatives at an organisational level through the notion of integration, itself composed of the dimensions of connectivity and freedom degree reduction. We conducted a comparative case study to compare the performance conditions of three patient committees (PFAC) in Belgium. Data were collected through observations, individual interviews and document analysis from 2018 to 2024.

**Findings:**

The cases presented three integration configurations that underlay the performance of each PFAC: strong integration, middle and weak integration. The combination of diverse forms of connectivity and reduced degrees of freedom significantly modified the PFAC’s capacity to perform.

**Originality/value:**

The systems theory approach provides tools for thinking about PPI in a way that complements analyses in terms of individual or organisational culture. The study helps to explore how to improve PPI initiatives deployed at the organisational level.

## 1. Introduction

Patient and public involvement (PPI) has been a major issue for health systems during the last decade. PPI generated great expectations such as enhancing health systems patient-centeredness or improving their quality (Scholl *et al.*, 2014; Wong *et al.*, 2020). The complexity and diversity of PPI initiatives have made it a central issue in the literature, grasped by multiple theoretical models (Tritter and McCallum, 2006; Carman *et al.*, 2013; Dent and Pahor, 2015). PPI initiatives take place in a multitude of forms at different levels of the health system such as care delivery (Guadagnoli and Ward, 1998), public policy and priority setting (Weale *et al.*, 2016), or organisation and quality management (Groene and Sunol, 2015).

Compared to primary care settings (Sharma et Grumbach, 2017; Liang *et al.*, 2018), hospitals seem to have jumped on the bandwagon rather late, particularly in western Europe. A cross-sectional study conducted in 2017 in four western European countries showed that, despite hospital actor’s motivation to embrace PPI, related initiatives still suffer from a lack of implementation (Scholtes *et al.*, 2021).

For years now, the literature describes a lack of evidence regarding the effectiveness of PPI initiatives and reporting on their impact (Crawford *et al.*, 2002; Mockford *et al.*, 2012; Lammons *et al.*, 2025). Evidence remains patchy due to the complexity of evaluating these initiatives. One of the main difficulties undoubtedly lies in defining what performance means for PPI. Indeed, PPI can emerge from empowering, consumerist or managerial logics (Martin, 2008; Voz *et al.*, 2021). Also, they may suffer from a lack of clarity regarding the general objectives underlying them or deviate significantly from them in their implementation (Martin *et al.*, 2018; Rowland *et al.*, 2021). While some studies are able to highlight certain successes, authors recognise that more needs to be done to understand how PPI can actually have an impact (Bombard *et al.*, 2018; Fredriksson and Modigh, 2021). Regardless of the logic behind PPI, the conditions for its performance must be determined.

Besides its rationales, PPI in quality improvement also varies in its forms. Among the most common forms are schemes where groups of patients are installed as members and regularly invited to sit on the board over a period of several years. In the United States, many hospitals have set up patient and family advisory councils (PFAC) (Herrin *et al.*, 2016). In France, hospitals have been legally obliged to set up user committees since 2012 (Pomey and Ghadi, 2009). In Belgium, PFACs emerged “spontaneously” in 2015 in certain hospitals, without any legal framework but with some incentives (Voz *et al.*, 2021). This paper seizes the case of PFAC in Belgium drawing on the statement that: “If public participation is to be transformative, then health care organization and management will need to create the conditions in which meaningful participation is possible” (Weale, 2016). Beside the call for PPI measurement (Oldfield *et al.*, 2018; Lewis, 2023), the paper aims to go beyond performance measurement to analyse the performance conditions of PPI initiatives at the organisation level.

## 2. Theoretical framework

We chose a particular angle of approach to the analysis to provide a useful complement to the analysis in terms of individuals (Liang *et al.*, 2018) or organisational culture (Renedo *et al.*, 2015).

In Luhmann’s theory, an organisation is a specific type of social system, described as a decision machine, where individuals participate as members in the organisational system (OS) (Nassehi, 2005; Seidl and Becker, 2006; Luhmann, 2018). Several subsystems operate within the hospital OS. The relationship between these subsystems is ordered according to different “forms of *differentiation*” which may coexist (Luhmann, 2021). Three forms of differentiation structure the hospital’s OS: vertical differentiation (or hierarchy, between the hospital management and the bottom, i.e. those whose work depend on the hospital management decisions), horizontal differentiation (or topography, between the operational centre, the carers and the periphery, i.e. all the support services) and organic differentiation (between the hospital’s different departments). Each of the OS’ sub-systems, such as the PFAC, is situated in the OS in relation to these three forms of differentiation.

The sub-systems that operate within the OS fulfil or participate in the fulfilment of a specific function, understood here as an internal effect, produced within a particular subsystem. Performance, meanwhile, refers to the external effects that a sub-system manages to produce in its intra-organisational environment (within the hospital), where the other subsystems of the OS operate (e.g. a surgery department or a quality and continuous improvement department). Effects come into play insofar as the *noise* of one subsystem becomes information for another (Luhmann, 2010). Our starting hypothesis is that the performance of a sub-system, in this case the PFAC, depends on its *integration* within the hospital’s OS. This integration can only be achieved through cooperation between the PFAC and the other subsystems of the OS.

Considering the relationships between OS subsystems implies that some form of communication can take place. We consider that communication is “not concerned with consistency, justifiability, truth or rationality but with connectivity” (Titscher *et al.*, 2007). The communication possibilities between sub-systems (their connectivity) condition their degree of integration within the OS. We will analyse the integration of the PFAC through two main dimensions. Firstly, the *expectations* vis-à-vis the PFAC. The question of expectations is essential here: “Anomalies, surprises, disappointments, all presuppose expectations in which they can appear (...). For this, internal preparation is essential, because irritations cannot be identified as such if they were not expected” (Luhmann, 2019). The second dimension is the way in which the PFAC reduces its *degrees of freedom* (its possibilities of action) to connect to the various sub-systems of its intra-organisational environment. These are the limits (in their functioning, in their work objectives, in their decisions) that PFACs agree to respect to meet the expectations of other subsystems, in the hope of being able to communicate.

## 3. Material and methods

Three case studies (Yin, 2009) of PFACs were conducted in French-speaking Belgium. The three PFACs monitored are based in three general care hospitals. Two PFACs were set up in 2015 with the third being launched six years later (Table 1).

**Table 1:** Characteristics of the three cases studied

	Case	1	2	3
Hospital characteristics	Type	*General care*	*General care*	*General care*
	Sector	*Private*	*Public*	*Public*
	Academic affiliation	*Non-academic*	*Academic*	*Academic characteristics*
PFACs characteristics	Year of creation	*2021*	*2015*	*2015*
	Hospital affiliation	*Quality department*	*Medical direction*	*Strategy management*
	Number of members (patient-professionals)	*10 (5–5)*	*19 (15–4)*	*9 (7–2)*
	Frequency of meetings	*Almost monthly*	*Monthly*	*Quarterly*

The fieldwork took place just over five years. Data collection was undertaken in three ways. Firstly, most PFAC meetings were observed between December 2018 and February 2024. Notes were taken of all observation sessions, including who was present, the subjects discussed, the content of the debates and certain exchanges. Secondly, a large amount of documentation was collected to compile the articles of association, minutes of meetings, activity reports and various formal and informal written documents. Finally, 32 semi-structured interviews were conducted with PFAC members’ and hospital professionals’ non-members of the PFAC (Table 2). Interviews dealt with the participant’s journey to the hospital and then to the PFAC, what the interviewee had to do there, and his or her experiences and expectations of the PFAC’s work within the hospital.

**Table 2:** Data collection summary

Cases	1	2	3	Total
Start of data collection	January 2023	December 2018	November 2018	
End of data collection	February 2024	June 2022	March 2022	
Number of interviews *(patient members – professional members – non-members)*	6 *(0-6-0)*	14 *(4-7-3)*	12 *(4-3-5)*	32
Number of observation sessions	5	12	6	23
Number of collected documents				More than 80

The aim of the analysis was to deepen our understanding of the performance conditions of the PFACs in the hospital, by comparing the three cases studied. After an exploratory reading of the material, all authors discussed the direction of the analysis based on its objective. An analytical framework, presented above, was developed by the authors to operationalise the hypothesis that the conditions for PFAC performance could be understood through the concept of integration. The first author then coded deductively using the two dimensions of integration for each differentiation form. The characterisation of each case, as well as the coding of specific situations, was discussed by the three authors.

## 4. Findings

Each case illustrates a different configuration of integration into the OS: a strong, a middle and a weak integration. The analysis does not examine the integration of PFACs in relation to the entire patient population, which is considered here as the environment of the OS (Voz *et al.*, 2024).

We depict briefly the results in Table 3. The following sections present details of the different configurations identified for each case through the analysis Supplementary Material. Case presentations begin with an overview of the case’s performance, so that readers can attempt to link the integration configuration with the actual performance of the PFAC. In these introductory remarks, the performance remains a perceived-one, as the objective of the methodological design was not to measure or evaluate this performance.

**Table 3:** Synthetic presentation of cases results

	Integration					
Differentiation	Vertical		Horizontal		Organic	
Integration dimension	Expectation	Degrees of freedom reduction	Expectation	Degrees of freedom reduction	Expectation	Degrees of freedom reduction
Case 1 – Strong integration	Strong expectations from direction	General limitations of functionings	No effective expectation from the centre	Strong RDF by a periphery service	Strong expectation from the Continuous Quality Improvement (CQI)	Strong RDF by the CQI department
Case 2 – Weak integration	Weak (and declining) expectations from direction	General limitations of functionings	No effective expectation from the centre	Weak RDF anywhere on the centre-periphery spectrum	Moderate and punctual expectations from diverse services	Weak and punctual RDF
Case 3 – Middle integration	Middle expectation from diverse direction	General limitations of functionings	No effective expectation from the centre	Middle RDF at the periphery	Acute expectation from one service and punctual expectation from others	Strong RDF by one service

**Source(s):** Authors’ own work

### 4.1 Case one: the strong integration

This case is the youngest but is also the one which seems to perform the best. It can extend its impact beyond the committee by contributing problem solving for the hospital. The following document extract gives an example of this:

Testimonial from a PFAC patient: “when I was first diagnosed, I was at a loss and had problems with the timing of the forms I had to fill in for the various reimbursements – I finally lost 8,000 euros!” As a result, attention needs to be paid to administrative support. CQI Director asks: “From an organisational point of view, what triggers the request for the social worker to visit patients to provide this administrative follow-up? This is a question to be reviewed with the nursing department. (…) This issue needs to be reworked internally (via the quality department) with the social worker coordinator” *Extract from the minutes of the plenary committee meeting, 20 April 2023.*

The PFAC’s performance takes the form of the selection and sharing of *a priori* peripheral or invisible information within the organisation, with the perspective of insuring that the patient flow is not delayed or complicated. As the CQI director said in an interview, “what it [the PFAC] produces are the themes that are addressed”. The PFAC’s performance lies in tackling issues that are usually left to one side, or that are far removed from the OS’s central concerns. The extract gathers and interrelates several subsystems which occupy diverse situations regarding to differentiation forms. We will see below how the PFAC performance relies on a strong integration in the hierarchical and organic differentiation, despite the integration at the periphery of the OS.

#### 4.1.1 Integration at the periphery of the horizontal differentiation

The core of the hospital, i.e. the healthcare professionals (HCPs), is nearly totally absent from the PFAC; the latter engaging much more with peripheral services and the support services. HCPs’ expectations of the PPI sub-system are very tenuous. The only time when the expectations of the core could be observed was in the months preceding the installation of the PFAC. Through a survey sent to all staff, HCPs had the opportunity to share their representations and expectations of what role the PFAC should play in the hospital. Several expectations were raised:

“We don’t want a patient union” or “Why should we have a PFAC, I already talk to my patients”, (…) We wanted to overcome these obstacles (*Interview extract, Care journey manager, PFAC member*).

A major expectation was visibly to keep the PFAC at a distance, in the periphery. This results in a great lack of visibility in “the field” as the care journey manager doubts the degree to which “the average nurse or secretary” is aware of what the PFAC does, or even than it exists. The degree of freedom remains consequently quite high, because it is impossible to reduce the PFAC’s degree of freedom in the absence of expectations.

#### 4.1.2 Strong vertical integration

The PFAC nurtures good connectivity with sub-systems fairly high up in the hierarchical differentiation. The strong expectations at the top of the organisation translates diversely. Expectations at the top of the hospital management are observed through its representation in the PFAC, as with the CQI director in the previous extract and in the regular participation of assistants to the medical and nursing directors. Even more importantly, these expectations are to be seen in the diffusion of the importance of PPI in the hospital, through bonding guidelines from the OS summit. For example, as our material shows, in documents as well as in diverse interviews, the PFAC justifies its activity through the connection of the PFAC and the axis of the strategic plan “guarantee an excellent patient experience rooted in the values of caring and empathy”, or through the inscription of the PFAC in the ongoing accreditation process. As several of the professionals interviewed said, senior management’s recognition (and diffusion) of the importance of PPI is an essential support in initiating partnerships within the OS. Nevertheless, the expectations remain relatively concise; for the summit, the essential is that a PFAC exists. Mainly, they expect basic organisational constraints, such as confidentiality, to be respected. Consequently, the freedom reduction is limited to these expectations.

#### 4.1.3 Multiple organic integration

Regarding the organic differentiation, the analysis reveals a dual position.

##### 4.1.3.1 Strong specific integration to the CQI

Firstly, the PFAC is strongly connected to the CQI department. Most of the professionals involved in the council are part of this department. However, as we will see in the other cases, professionals taking on a role in the PFAC is not sufficient to ensure strong integration.

Muriel, who takes care to listen to patients as she constructs her itineraries, taking into account their experiences in terms of protocol and the trajectory of care. And then there’s therapeutic education, with Madeleine. And Laure who is the quality improvement coordinator with Mr Gilles. So, the four of us have a vision of what we have built up, which is called the patient as partner, the patient as actor in his or her own health. We can list several ways of looking at patient participation, and the patient committee [PFAC], is one way of looking at it Interview, PFAC and humanisation of care coordinator.

As the extract shows, the connectivity to CQI department is built on a strong expectation, which takes place in a collective “vision” vis-à-vis the PFAC. Each listed professional used to work directly with patients, or patient opinions, in their pursuit of quality. The deployment of the PFAC is part of an organisational context in which this sub-system of the OS, for its own purposes, regularly seeks to “listen” to its user environment; this is a work process already established, by other means, for the CQI sub-system. In this way, the CQI department has high and precise expectations about the PFAC: the latter has to make available to users the patient’s observations of the connectability to diverse hospital services. The practice feeds into the work of professionals in several areas: support for people with special needs, review of clinical itineraries or therapeutic education. In these domains, their attention is already focused on the connectability of the hospital services, just as the PFAC is expected to; that is to say, the degree of appropriate and uncomplicated usages of concerned services. These expectations of the CQI correlate with strong freedom degree reduction, as the following extract introduce:

It’s important to us that our patient partners are trained and informed about their role, and that they are involved in building and not destroying (…). That’s not the aim. We need to move forward together (...). This means that we are aware of the requests [from professionals] and we can sometimes clarify certain things by saying “Yes, you can ask for that, you can’t ask for that, that’s not the purpose of the PFAC”. In general, we ask the professionals to come and present things themselves. And it goes rather well *Interview extract, professional member, quality coordinator.*

The PFAC constrain itself significantly to meet the CQI’s expectations. It starts by representing users the “right way”, through training, information and accumulated experience from sharing in PFAC’s meetings, as well as the oft-repeated importance of appropriate recruitment (in which CQI professionals are directly involved). Objects of discussion, guests, agenda, are mainly decided by the CQI professionals. As one patient pointed out at a meeting, compliance with these expectations can take some time:

We became partners, without suffering the committee. At the start, the structure was new to us, we were observers, and it took time to evolve in our posture (Meeting observation, 19 January 2023).

PFAC members model themselves to meet the CQI’s expectations. As the previous interview extract shows, CQI professionals also prepare OS’s sub-systems to meet in the PFAC. We will see in the section below how the strong organic integration in the CQI department enables occasional connectivity with other sub-systems. The link between the two sub-systems, the PFAC and the CQI department, reinforces the legitimacy of the CQI’s actions: just as the President of the Medical Council is legitimised by his doctors, the CQI is legitimised by its users. It permits a wobbling organic integration with other sub-systems.

##### 4.1.3.2 Mediate connectivity with other services

Building on the strong hierarchical integration and the strong integration in the CQI department, the PFAC can connect regularly with other sub-systems of its environment, without the one-off nature of these connections having any negative consequences. This secondary integration is both essential and totally dependent on the work of CQI department. It is essential because it is through this connectivity that the PFAC can observe diverse facets of the OS. It is totally dependent on the work of the CQI department because of the mediation interplay the latter supports. During the whole observation period, no connectivity occurred without the intermediary of the CQI department. When the integration to the latter is strong and continuous, integration to other services can be occasional. Indeed, expectations of other sub-systems occur only relatively infrequently to specific projects. Thus, the generation and nurturing of expectations in the OS is a central issue for the PFAC and the CQI department:

You can feel that they [PFAC members] are gradually gaining in maturity and experience. So, the further they go, the more we might have members who could then join a hospital’s first official committee. At the same time, we’re continuing to prepare all the professionals through the accreditation process (...) and they’re even starting to think about it, to say to themselves: “Hey, we could really use a patient in this project”. *Interview extract, CQI Director*.

This director’s reflections are illustrative of the mediation interplay that the CQI department operates. Partly because of the recent history of this PFAC, mediation is still necessary, until members are more “mature and experienced” and professionals more “prepared”. The spontaneous enunciation of the possible “use” of a patient is already an achievement for the director, as it promises increasing connectivity in the future. These emerging expectations remain quite low and punctual. In the same way, having a PFAC member joining “a hospital first official committee” would ensure more permanent and less mediated connectivity. For the PFAC, strengthening these links is a central issue in its existence. In addition to the *de facto* links already established by the composition of the PFAC, as well as those that are forged on an *ad hoc* basis with certain departments during specific projects (e.g. the review of an information brochure for medical imaging), the PFAC is seeking to create structural relationships with more independent sub-systems and high-hierarchical sub-systems such as the Board of Directors or General Management.

### 4.2 Case 2: unidentified performance and chronic disintegration

This PFAC is the oldest among our cases. Its history is marked by the fact that it was brought to a close during our observation period due to a lack of performance. In this case, the performance still appeared to be impalpable. After the initial enthusiasm, the PFAC’s performance was regularly questioned, by its members (“if I resign from the PFAC one day, it will be because of its lack of influence”, *patient member interview extract*), or by those outside the PFAC (“when the new manager arrived, he asked us ‘what is it for?’”, *medical director interview extract*). The following extract approaches a central problem for this PFAC:

And they [patient members] say it, but we don’t hear it, the lack of humanisation in care, the lack of information, the lack of consideration (...) the lack of follow-up, but they don’t say it in a structured way, it’s just complaints *Interview extract from head of psycho-social department, professional PFAC member*.

The extract reveals a salient fact about this PFAC material: many things are said that the organisation “doesn’t hear” (complaints are noise, cf. supra). If the interviewee feels that the professionals or the hospital, “we”, are not hearing the PFAC, we argue it is an organisational connectivity problem, not an individual one. In the same way, if the intuition of a lack of structure seems promising to us, it must certainly relate more to the structure of reception than of expression. We will see below how this case is marked by a weak integration configuration.

#### 4.2.1 Horizontal non-integration

Towards the centre-periphery differentiation, this case is marked by a kind of ambivalence which complicates its performance possibilities. Indeed, a physician has been appointed as a member of the PFAC, and there is a formal link with the medical direction. These elements could give the impression of strong integration at the centre. Despite this, the PFAC struggles to connect with the centre of the OS, as the following material shows:

One of the members suggested setting up a discussion group in the diabetology department. The coordinator explained that the hospital authorities had recently refused to set up activities with volunteer patients. They did not want patients to be placed in a one-to-one discussion with others, with no control over what was said. Observation, working meeting, 12th February 2019

The observation notes reveal the OS’s expectation towards the PFAC: keep a distance to the centre, the therapeutic activity. On several occasions during our observations, PFAC members promoted the idea of engaging with patients about their pathology or clinical experiences. Each time, the idea was shelved. In the same vein, patient members complained that their request to meet medical services managers was refused, which a professional member replied:

It [the request] was not challenged, but the question was what your objective was beyond just a physical meeting (Observation, Plenary meeting, 24th September 2019).

This question has never been answered. It illustrates rather well the absence of expectation from the centre – as we saw earlier. Consequently, on the rare occasions when the centre has been encountered, it seemed to produce nothing but a discussion without orientation.

This situation results in the impossibility for the PFAC to situate itself in the centre-periphery differentiation. Indeed, the connection with the periphery is not clear either – as no specific sub-system seems to have expectations towards the PFAC. It posits an existential problem for this sub-system, concretising on regular questions concerning its aims and missions. This situation of disintegration is much more problematic than if the PFAC had been integrated into the periphery, which did not seem attractive at first sight.

#### 4.2.2 Declining integration to vertical differentiation

The PFAC suffered from a declining connectivity with the summit of the OS, resulting in a weak integration into this form of differentiation during our observation period. Several elements in our material attest of this situation. The origin of the PFAC lies in a request made by the hospital’s Chief Executive Officer to one of the Medical Directors to set up this body. Since then, the committee has been formally linked to medical management via the organisational chart. Unlike the previous case, none of these directors is directly involved in the project, apart from receiving an activity report or approving internal regulations. Hospital representatives who attend the PFAC meetings, as well as those who endorse coordination positions of the PFAC, are drawn from “the hospital mediation department, medical staff, nursing staff and the clinical psychology and social action department (...)” (extract from the internal rules) and are not occupying positions in the OS top management. Moreover, while the early days may have aroused the interest of the medical and communication managers, the departure of the first PFAC chairman undermined these expectations. Consequently, the PFAC connected very weakly with the summit of the OS during our data collection period.

#### 4.2.3 Instantaneous and diffuse organic integration

When looking at the organic integration of the PFAC, our material shows that it is characterised by its wide distribution and its instantaneousness, which explains the unmediated relations with other sub-systems. Unlike in the previous case, this PFAC is almost directly connected with the OS’ surrounding sub-systems. Although requests go through an e-mail address managed by the PFAC manager, they are not processed in any way before they reach the PFAC. They are relayed directly. Once again, connectivity might therefore appear to be strong, or at least easy, between the PFAC and the OS. However, we shall see below that this causes a fundamental problem of adjustment.

We first look at the diffuse nature of the connectivity, by means of two indicators. Firstly, hospital representatives formally link the PFAC to a large panel of hospital services. But, the connectivity of the PFAC is not enhanced by the presence of these professionals in the PFAC, as they seem to share no precise expectations and give too much freedom to the PFAC. Hospital mediation, psychosocial services, medical and nursing staff are all engaged by one of these professionals. None of the representatives took advantage of the observations shared in the PFAC meetings to either feedback information to their respective sub-systems or to communicate with other sub-systems. Secondly, the PFAC has faced an incredible amount of diversity in the received requests, which prevents it from constraining itself:

The PFAC was able to pursue several activities (...): Proofreading of online PRIM questionnaires. Nephrology department project: setting up a group of patient partners to improve care (…). Taking part in a panel to test waiting room chairs. Student dissertation project into the patient partnerships in adult oncology departments. Request for patient participation in the development of a guideline for occupational therapists with a view to maintaining the functional capacity of elderly (…) living at home. Participation in a working group to consider future registration desks with regard (…) to fit protective glass. Belgian Cancer Barometer: consultation of online questionnaires on patients’ experiences *Extract from the 2020 activity report*.

In each of the activities listed, the PFAC acts as a pool of available patients, solicited by a wide variety of sub-systems, sometimes even from outside the hospital. The understanding by the surrounding subsystems of the PFAC seems to be that of direct access to *their* specific patient group of interest. Their expectations rely on the possibility of an easy access to a representative sample of their population of interest, which is always more specific than the general user composition of the PFAC. Connecting in this way does not allow the PFAC to comply with the subsystems’ expectations.

The diversity of these requests brings us to the second characteristic of the weak integration: its instantaneousness. Indeed, all the demands made to the PFAC required a one-off connection between the subsystems. Due to this temporality, the PFAC never had time to really adjust itself, to reduce its degree of freedom, to meet the others’ expectations. Symptomatically in our material, a major point of tension for several members of the PFAC was obtaining feedback after their contributions – something that was always almost impossible. Given this situation, the future understanding of expectations as well as the adjustment of the PFAC functioning was complicated. No memory of the subsystem could be constructed to feed into future decisions.

### 4.3 Case 3: situated performance and wobbling integration

The third case undoubtedly occupies an intermediate position between the first two. Most of the people we met were globally pleased with the PFAC’s performance. The PFAC operates as its statutes intended:

By the participation of patients of the PFAC in certain working groups and/or institutional committees; and by the organisation of regular exchanges between the PFAC and the hospital *Extract from Internal Rules*.

Moreover, several PFAC achievements can be directly identified in the few years it has been in existence, such as “the patient checklist”, a guide that was produced to prevent familial, social, financial difficulties due to hospitalisation. However, besides those few concrete achievements, performance remains quite intangible, as the following observation note illustrates:

Today’s meeting was dedicated to several consultations of PFAC members regarding: the colour of nursing staff uniform, the presence of hydro-alcoholic solution in bedrooms, the project of patient-resources. Each presentation elicits a few reactions, with no way of knowing what will become of it. At the end of the meeting, a patient shared concerns about the availability of chairs for the accompanying persons. The coordinator reacts “I will relay it, not only in the minutes” *Observation notes,* *22nd* *November 2018*.

While participant’s statements seem to attest to a good performance, it is harder to see what has been achieved when we analyse the material. Given the number of contacts between the PFAC and the hospital there seems to be relatively few concrete achievements. We will see below how this situation is built on a middle integration of the PFAC in the OS.

#### 4.3.1 Strong horizontal differentiation integration at the periphery

The centre of the OS is clearly quite distant from the PFAC. Unlike the previous case, no data collected reveals any direct connection between the PFAC and any HCP representatives. Beyond the discourse that the PFAC could “speak to everyone”, the observation shows that it has something to say mainly at the periphery of the OS. Even when HCPs participate in the PFAC, it is not as a physician or a nurse but as project promotors or a staff manager. When they sit at the PFAC, it is hardly ever to speak of therapeutic activity (the centre) rather more to present support activity of the OS (the periphery). Symptomatically, the interviewees spoke of their doubts as to whether HCPs were familiar with the PFAC. But, despite the mention of initiatives to raise the visibility of the PFAC, this apparent ignorance does not emerge as a problem for the PFAC connectivity – as there is no effective expectation at the centre.

Because sometimes when I try to talk about our reception practices with colleagues in the care units, they’ll practically say “Well, yes, but … reception is your job, it’s your area, you’re responsible for reception, I’m responsible for care”. (...) So every time we come up with an idea, we have to test it out, really test it out with the PFAC to see whether it represents the majority and whether it’s essential or not *Interview extract, Patient Administrative Services coordinator*.

The extract reveals a central characteristic for this PFAC. As in other cases, the centre keeps a safe distance from anything it considers to be outside its field of interest. Consequently, peripheral themes are dealt with by periphery professionals, with an expectation to “test out” their ideas. As for the first case, this trend is confirmed by the professionals who take part in the PFAC on a regular basis. The coordinator is from the Quality and Strategy department, the hospital ombudsman joins in almost every time, and the person in charge of Patient Administration Services participates from time to time. Once again, this integration at the periphery, rather than at the centre, is not a problem for the PFAC – and it is a common feature of the three cases. We will see in the next section that the way it integrates in the hierarchical differentiation certainly poses more problems.

#### 4.3.2 Weak vertical integration

The hierarchical integration of this PFAC is quite different from the two previous cases. Formally, the PFAC is included in the hospital’s organigram as an “independent” advisory body. It has no dependency or hierarchical links identified in the organigram. The PFAC maintains a direct relationship with middle management professionals, from which the coordinator, as well as the frequently visiting professionals, comes from. The only regular link between the management teams and the PFAC is through the sending of the minutes to all the directors, who said in interviews that they were going through these reports. We see this way of interacting as problematic, as the following excerpt shows:

The aim is also, and above all, to enable patients to express their views on what is going well and what is going less well, to be able to refer these elements to management and for management to decide whether or not to “OK, we’ll decide to implement actions” Interview extract, committee coordinator.

If it is to have any impact, the PFAC must be able to feed into the decision-making process. However, decision-making capacity resides in places to which the PFAC can only “refer” by transmitting the meeting minutes. In the absence of more direct involvement of management in the PFAC, the latter struggles to reduce its degree of freedom, and to respond favourably to the management’s expectations. Moreover, we have observed that the most directly observable effects were produced when they related to issues over which the coordinator had a “free hand” – which are less numerous for a middle manager.

#### 4.3.3 Strong and specific organic integration

The possibility of communication is always established with the PFAC coordinator, i.e. a professional of the Strategy and Quality Department. No PFAC meeting is held without her. The Strategy and Quality representative also manages patient satisfaction surveys, among other things. Communication between these two sub-systems is made possible by the tendency to learn from differences that have been observed elsewhere. The process is already mobilised by the department representatives. There is a strong connection with the coordinator for whom great expectations permit the PFAC relative freedom albeit within a restrictive framework.

It is also through the coordinator that most of the possibilities for connectivity with the hospital’s other sub-systems are opened-up. Observations show regular connectivity with other hospital subsystems. Some come to the PFAC on a “regular” basis, while others make occasional or even one-off appearances. Among the units that regularly connect with the PFAC, we noted several communications with the “HR, Administration and Patient Transport” departments, and more specifically with its division in charge of patient administration. Communication is established “on demand”, when the sub-system she represents deems it necessary, and permitted by clear and limited expectations. The PFAC’s connectivity is therefore particularly dependent on demand from surrounding subsystems.

Following a presentation on an oncology support centre, a patient suggested that dietary workshops should be organised as part of preventive care rather than curative care. The coordinator suggested that a day be organised at the initiative of the PFAC on the theme of “healthy eating for everyone”, not in the context of illness, but in the context of prevention and health promotion. The members were all in favour of organising this day in 2020 (…). Jeanne also suggested that the dietetics department take part. Coordinator will check with management to see if this is feasible and possible. Extract from Minutes, 11 September 2019.

The situation described above is typical of the moments of connectivity mediated by the co-ordinator. Indeed, common sense might lead us to expect that the connectivity between the PFAC and a particular hospital sub-system would produce “useful” communication with the latter. We see here that this is not the case. It is only because the coordinator takes on the role of observer that something is produced. And the addressee is rather her own pole than the subsystem that has come to present a project. The dependence on the specific organic integration is a problem for connectivity in several situations. Particularly, this problem reveals in situations where PFAC members have to connect directly with surrounding subsystems, whereas an interviewee said:

They don’t talk much, but at the last meeting we asked: “Do you have anything to add? And that’s it … Now, sometimes the subjects are so … At our last meeting, we were discussing the way in which all medical alerts are communicated between physicians. It’s true that she perhaps didn’t have much to say … We might have discussed the way in which private rooms were allocated within the hospital, she might have had something to say, that’s all … ”. Interview extract, Chairman of the Medical Board.

Direct connection with surrounding subsystems often produces very poor results. No achievement has been raised in the material in these situations. Indeed, expectations are not clear from the subsystem, which does not permit the PFAC to comply in a relevant way.

## 5. Discussion

As has been shown in other contexts, “voice is not enough” (Satterstrom *et al.*, 2024). To ensure that a genuine form of participation can take root within hospitals, we wanted to understand what makes it work, not only within the PFACs deployed but also in the way in which the ideas produced can percolate through the SO (i.e. the performance). The analysis presented in this article deliberately leaves aside the debate surrounding performance objectives. Our analysis does not engage in a value-laden discussion about whether PFACs performance is “good” or “bad”. Rather, it seeks to explain what could enable PFACs to be effective, based on a very value-neutral understanding of what it means to be effective: to have an (unqualified) impact within its intra-organisational environment.

The results revealed that differences in the forms of differentiation of the OS’ subsystems strongly influence the performance potential of PFACs. We draw three main lessons from this: a strong desire for vertical differentiation is necessary, an integration at the periphery of the SO does not prevent the PFAC from performing, and that a specific organic integration seems more profitable and allows mediation, as opposed to a diffuse organic integration. In our opinion, the results of this performance analysis raise a challenge and suggests ways to overcome it, for the implementation of PPI: by reducing the gap between formal and functional integration of the various initiatives within hospitals.

First, the results show that establishing a formal link with a directorate, without concrete commitment, is not enough to make the PFAC effective. Conversely, the strongest case of integration occurs where a director sits on the PFAC. Integration is weakened as the concrete commitment to the scheme diminishes. Previous studies regularly point to the need for management commitment, as reported in a systematic review of the literature (Bombard *et al.*, 2018). A quasi-experimental study also highlighted the importance of leadership and commitment at all levels of the organisation for the success of public participation in decision-making processes (Abelson *et al.*, 2007). Our results are numerous in this sense and give concrete content to the notion of management commitment, which must go beyond mere formal support or support in principle. The management linked to the PFAC must make a concrete commitment to it and implicate it when carrying out its own work.

Second, the analysis showed the high importance of the expectation dimension of the connected subsystems, as a prerequisite for reducing the degrees of freedom of the PFAC. The notion is often used referred to as “common sense” in the literature. For example, a success strategy identified by PFAC players is “clear alignment with and line of sight on organizational quality” (Hatlie *et al.*, 2020). Yvonne Bombard’s systematic review indicates that several studies mention the need to “clarify the objectives, roles, and expectations of the engagement for patients/carers” as a central “technique to enhance design of engagement” (Bombard *et al.*, 2018). It is worth noting here the extent to which it appears to be a necessary condition for any form of integration. The results show that *a priori* and *in itinere* work on expectations can condition the performance of such initiatives. More in-depth work on expectations would undoubtedly make it possible to ensure a better context-method fit (Abelson *et al.*, 2007) and to prepare for more appropriate evaluations of schemes (Boivin *et al.*, 2018). In many cases, there is probably no reason to regret the lack of clinical impact, or proof of impact, when the real expectation was not in that sector. It brings up the notion of “situated expectations”, which is directly related to the next point.

Third, a reflection on the autonomous nature of this type of system deserves to be held in light of our results which argue for greater integration within the organisation. To remain an operational system, it must remain autonomous. But, if we want it to perform, it must not remain independent. Its autonomy is the condition for the interdependence, and therefore the integration, of the PFAC. The second case, which could appear as the most independent, is also the one that will perform the least well. An analysis in terms of power might suggest that PFACs need to be independent in order to act as a counterweight. We believe that if they are to perform well, they must be an integral part of the OS; within which they are situated. Our results show that it is sometimes better to be integrated into a very specific department of the hospital than to have the impression that there is a possibility of connection with the whole hospital. In our view, this finding is in line with what has already been observed in other studies, namely that PPI activities based on several professionals already juggling a number of other responsibilities lead to token participation and limited results (Gagliardi *et al.*, 2021).

## 6. Conclusion

If PFACs are the “bedrock of healthcare quality” (Unaka *et al.*, 2022), researchers must undoubtedly be able to provide guidance to hospital teams to optimise their implementation. We believe that the results and ideas shared will be useful in helping to shape the design of future PPI initiatives of this type. They will need to strike as close a balance as possible between formal and functional hospital integration.

## Supplementary Material

Data supplement 1

## Notes

The supplementary material for this article can be found online.
