# Supplementary material for: Managing integration and disintegration: performance conditions of patient and public involvement to hospitals quality improvement
Source: J Health Organ Manag. 2026 Apr 2;40(9):228–42. doi: 10.1108/JHOM-10-2024-0410 (PMC13053202; doi:10.1108/JHOM-10-2024-0410)
Supplement: Data supplement 1 [file jhom-10-2024-0410_suppl1.docx]

**Supplementary file – Additional material**

As with the manuscript, all names have been replaced with fictitious pseudonyms.

**Case 1**

*Performance*

« We also presented them with the complaint management procedure, which was an accreditation requirement. We have a procedure, but it had not been developed in collaboration with patients. So, we presented our procedure to them so that they could provide their feedback and comments, and we adapted it so that we could then present it to the accreditation and implement it ». Interview extract, PFAC professional member.

« Continuation of internal management of the issue of home hospitalisation:

- Jack proposes to return with Marie-Hélène to present the contents of the home hospitalisation project patient satisfaction survey to the patient committee.
- Return to management to demonstrate the PFAC's interest in discharge culture in general.
- Organise a focus group with patient partners on their priorities: what constitutes a successful discharge? What constitutes a failed discharge?
- For the internal quality department: focus on ongoing internal projects at other sites that are moving in the direction of this type of coordination for patients returning home. Write up best practices and submit them to the patient committee » Extract of the activity report 2021-2023.

« At the second or third meeting, we were talking about staff and the training plan. One of the patient partners said at one point, ‘In any case, you should have training for your staff on communication between professionals and communication between professionals and patients.’ So we discussed it with human resources and set up the training. So when we come back to them and say, ‘Yes, we took it into account and set up this training,’ that's why I say it works well. They are also heard, we are able to communicate the information ». Interview extract, PFAC professional member.

*Mediate connectivity with other services*

« Well, we can see that they are gradually gaining maturity and experience. So, the further they progress, the more likely it is that, at some point, we may have members who could then join the first official hospital committee. At the same time, we are continuing to prepare all stakeholders through the accreditation process ». Interview extract, CQI director.

« And so, they can move forward with this work. But for me too, in terms of coordination, it's about bringing the HCPs and them together. Everyone wins: the HCPs, because there is normally a benevolent attitude, because I work to ensure that they are benevolent here, which means that they are grateful for their activities. And at the same time, we integrate otherness and an outside perspective. And then we can see if we are creating the conditions so that what has been identified by the patient partners is not a criticism but an improvement » Interview extract, PFAC professional member.

« So, we have a request form, which is what we'll be presenting tomorrow, and a request form where the stakeholder specifies, ‘I'd like to create a new brochure with the patients’ or ‘We wanted to review...’ ‘Well, it's the same thing again...’ ‘I'd like to review the structure of my department,’ ‘Well, okay.’ Or, ‘We have a small space dedicated to patients and their companions, for example, in paediatrics. We would like to make it a little more user-friendly. We would like to do this with parents or other people involved.’ So, normally, they have a form. They send us the request saying, ‘I'd like to do this, I need two or three patients, I'd like them to have this profile, etc.’ We at the office look at their request, analyse it and provide guidance » Interview extract, PFAC professional member.

*Strong vertical integration*

« PFAC meeting of 1 February 2024

1. Welcome and introduction of new members.

[Present] to welcome new members to the PPC:

Michel Champlain, Hospital Site Director, and Nadine Gagnon, Quality Improvement Coordinator » Extract of the presentation material for plenary meeting, 1 February 2024.

**Case 2**

*Performance*

« It's like a dog chasing its tail. So... We don't want to maintain a structure that serves no purpose and causes dissatisfaction. And that's kind of how I feel coming out of the crisis. So they asked to meet with me to discuss it. They were upset because, coming out of the crisis, we discussed ROI and switching to volunteer contracts without talking to them about it » Interview extract, hospital director, non-member.

« We know what a works council is, but we don't know what a PFAC is, so everyone experiences it differently and everyone has their own vision, fears and fantasies. For me, it was a forum for consultation between carers and patients, with a view to improving the quality of care and therefore the quality of life of both HCPs and patients. For me, that's what it was! And sometimes, our PFAC functioned like that. When they challenged us on... not everything is negative... on the delay in registering with the Health Network, when they challenged us on parking, on registering with the polyclinic... all of that was interesting » Interview extract, PFAC professional member.

« We have put patient representatives in the working group for this care journey, and if possible, patients who were involved in the cancer patient's journey. (silence) But ultimately, we wonder whether interviewing people in the waiting room a few times might not be more effective » Interview extract, hospital director, non-member.

*Declining integration to vertical differentiation*

« I honestly think it was created at a time when it was fashionable. (...) And the notion of patient participation, or citizen participation, is a trend of the 21st century. And I think that, initially, there was no expectation » Interview extract, hospital director, non-member.

« I feel that this patient committee was set up because it was fashionable, and because hospital gold standards require a patient committee. But even though things are changing, based on my interactions, I still feel that from the outset, this committee was set up without any deep reflection on the role of a committee within the hospital system. Ultimately, I still feel strongly that this was a case of exploiting the concept. (...) I'm not sure that our healthcare professionals and hospital managers are cognitively aware of the need for a paradigm shift. So we do what we have to do, and often pretend a little. (…) That doesn't mean that we're integrating it into our structures on the ground. We're adapting to it in order to survive. But we haven't integrated it » Interview extract, PFAC professional member.

« I've been head of department here for several years now. And then they picked on me... Why me on the PFAC, eh? (...) Listen, I don't go there much anymore because of that [the conflicts], then they complained because I wasn't coming anymore, so I kind of had to go, but it drains me, it really drains me to go there, it really drains me to go there” Interview extract, PFAC professional member.

*Instantaneous and diffuse organic integration*

« Dear Ms Wolfe,

First of all, I would like to thank you and the entire patient committee for taking the time to review this draft brochure as part of our project to welcome families to the intensive care unit.

We will be sure to make the various suggested changes so that the information is as clear as possible for patients' loved ones.

Have a great day and thank you again!

Kind regards,

Franz Fredrik

Head Nurse
General Intensive Care » Excerpt from 2019 committee email correspondence

« Dear Ms Tremblay,

I am currently writing brochures for patients in the medical oncology department.

These brochures cover treatment regimens and managing the side effects of these treatments. They therefore contain a lot of recommendations.

Currently, the brochures are written in the imperative ("Take your medication on an empty stomach", etc.). Some of the people who are proofreading these brochures have pointed out to me that this wording is very directive.

I have therefore been advised to contact you to find out what the patient committee thinks about this. Is it better to use ‘Take your medicine on an empty stomach’ or "Take your medicine on an empty stomach" or does it not matter?

Thank you in advance for your help.

Kind regards,

Madeline Dicenzo» Excerpt from 2019 committee email correspondence

**Case three**

*Performance*

« If we believe that it is a valid concern and that it is not just one patient's complaint but could be the complaint of several patients, or even the majority of patients, we discuss it with management and see what we can do to improve the situation, that's for sure. In any case, I take minutes of each meeting, send them out, they read them, and sometimes I am contacted again to say, ‘We have asked Mr So-and-so to see what can be put in place’. So I know that things are being done. I'm not saying that everything has changed, but in any case, some things are changing. It's in our best interest, I would say. That's the goal, too! To be able to see where things aren't going very well and improve them » Interview extract, PFAC coordinator.

*Weak vertical integration*

« My colleagues don't read the minutes, and I could say to myself, ‘OK, they have other things to do,’ but for me, it's part of my job, so yes, I read them. If there are any points of concern or if I see that there are issues that seem to be getting stuck, I alert one or other person internally to say, "Hey, I read in the minutes committee that... do you want to contact the coordinator to see what's going on and get things moving?" This has happened to me several times... mobility or something else. Now, the coordinator gives me regular feedback » Interview extract, CQI director.

« I give them a week [at PFAC, to review the minutes] and then I send it to the board of directors. But it's rare that I get any feedback. But they read it because they say ‘received’...» Interview extract, PFAC coordinator.

*Strong and specific organic integration*

« And yes, I think the coordinator guides them well... ‘guides’ in the sense that she also knows how to calm things down a bit sometimes, she knows how to tone down the rhetoric a little... You shouldn't take everything that is said at the patient committee meetings at face value either (...) I think that by putting things into perspective, it doesn't bother me. And as I said, there is a debriefing afterwards where we analyse what was said and try to get to the heart of what was said and what we are going to work with » Interview extract, middle-management professional, non-member.

« Yes, I transpose. It's true that I may be led to transpose something very personal into something else. Because there's the patient committee, but there are also patient satisfaction surveys, and I'm responsible for those. We have a complaints department. So you have opinions and things expressed by patients who come from different backgrounds, and you end up grouping them together too. And you make connections between these different elements, which allows you to say that it's not just one person expressing it, but several, and so it's not just the voice of one patient, it's the voice of a few patients, or even many patients. So what do we do with that? It's really a gathering. For me, it's extremely important, in the patient's entire experience, to have not just one method » Interview extract, PFAC coordinator.
